# Unfaithful Maintenance of Methylation Imprints Due to Loss of Maternal Nuclear Dnmt1 during Somatic Cell Nuclear Transfer

**DOI:** 10.1371/journal.pone.0020154

**Published:** 2011-05-20

**Authors:** Yanchang Wei, Yanjun Huan, Yongqian Shi, Zhongfeng Liu, Gerelchimeg Bou, Yibo Luo, Li Zhang, Cairong Yang, Qingran Kong, Jiangtian Tian, Ping Xia, Qing-Yuan Sun, ZhongHua Liu

**Affiliations:** 1 College of Life Science, Northeast Agricultural University of China, Harbin, China; 2 State Key Laboratory of Reproductive Biology, Institute of Zoology, Chinese Academy of Sciences, Beijing, China; 3 Department of Obstetrics and Gynecology, School of Medicine, University of California Davis, Sacramento, California, United States of America; Institute of Zoology, Chinese Academy of Sciences, China

## Abstract

The low success rate of somatic cell nuclear transfer (SCNT) in mammalian cloning is largely due to imprinting problems. However, little is known about the mechanisms of reprogramming imprinted genes during SCNT. Parental origin-specific DNA methylation regulates the monoallelic expression of imprinted genes. In natural fertilization, methylation imprints are established in the parental germline and maintained throughout embryonic development. However, it is unclear whether methylation imprints are protected from global changes of DNA methylation in cloned preimplantation embryos. Here, we demonstrate that cloned porcine preimplantation embryos exhibit demethylation at differentially methylated regions (DMRs) of imprinted genes; in particular, demethylation occurs during the first two cell cycles. By RNAi-mediated knockdown, we found that Dnmt1 is required for the maintenance of methylation imprints in porcine preimplantation embryos. However, no clear signals were detected in the nuclei of oocytes and preimplantation embryos by immunofluorescence. Thus, Dnmt1 is present at very low levels in the nuclei of porcine oocytes and preimplantation embryos and maintains methylation imprints. We further showed that methylation imprints were rescued in nonenucleated metaphase II (MII) oocytes. Our results indicate that loss of Dnmt1 in the maternal nucleus during SCNT significantly contributes to the unfaithful maintenance of methylation imprints in cloned embryos.

## Introduction

Somatic cell nuclear transfer (SCNT) has been successful in a variety of species [1], [2], [3], [4], [5]. These reports indicated that differentiated somatic cell nuclei can be reprogrammed to totipotency when transferred into enucleated oocytes. However, the success rate of SCNT remains low, frequently because of imprinting problems [6], [7].

Genomic imprinting is an epigenetic mechanism that ensures parental origin-specific monoallelic expression in certain mammalian genes. The imprinted genes play essential roles in embryonic development, postnatal growth and adult behaviors [8]. The developmental failure of uni-parental (bi-maternal and bi-paternal) embryos has indicated the functional importance of imprinted genes in normal development [9], [10]. Mouse migrating primordial germ cells (PGCs) at 8.5 to 9.5 days postcoitum (dpc) can be successfully used as donors for nuclear transfer, whereas gonadal PGCs at 11.5 dpc and later are incompetent to support full-term development [11], [12]. Together, these findings suggest that proper imprinting is highly correlated with the developmental potential of cloned embryos.

Methylation imprints are established during germ cell development and are protected from genome-wide demethylation and re-methylation in early development [13]. It is of interest to understand whether methylation imprints in donor somatic nuclei are protected from the global changes of DNA methylation in early embryos as effectively as in fertilized nuclei. In fact, cloned animals frequently exhibit abnormalities (placental and fetal overgrowth and perinatal death) that typically result from deregulation of imprinted genes, perhaps indicating that SCNT might cause aberrant imprinting patterns [14]. A study of cloned mice revealed that some imprinted genes (and also some non-imprinted genes) were abnormally expressed in cloned mouse embryos [15]. An abnormal allelic expression pattern of the imprinted *Igf2r* gene was also found in cloned bovine calves [16]. Cloned animals frequently exhibit abnormalities that resemble these diseases in human imprinting diseases and in imprinting gene experimentally mutant mice [17]. Increasing evidence supports the hypothesis that maintenance of methylation imprints is ineffective during SCNT [6], [7], [18], [19].

Although the role of Dnmt1 in the maintenance of methylation at imprinted genes in post-implantation embryos and somatic cells is well established [20], there have been conflicting and puzzling results related to how these imprints are maintained in cleavage-stage preimplantation embryos [21], [22], [23], [24], [25]. Previous findings suggested that Dnmt1o (oocyte form) was localized within the nucleus for only one cell cycle during preimplantation development and that Dnmt1s (somatic form) was undetectable in the nucleus. This seemed to exclude the possibility that Dnmt1 could be an imprinting maintenance enzyme functioning during preimplantation development [21], [22]. However, two other studies have argued against these earlier findings, and suggested that Dnmt1s could indeed be involved in the maintenance of methylation imprinting in cleavage-stage preimplantation embryos [23], [24]. Using antibodies specific for Dnmt1s, both studies revealed that the somatic form of Dnmt1 is present at very low levels in the nuclei of preimplantation embryos (approximately 1/2,000 of the total cellular Dnmt1). The major form of Dnmt1 is Dnmt1o, which is mainly localized in the cytoplasm [23], [24]. The presence of Dnmt1s in the nuclei of preimplantation embryos again presents the possibility that Dnmt1 could be involved in maintaining the methylation of imprinted genes. Finally, Hirasawa et al. (2008) found that knockout of *Dnmt1* (both oocyte and somatic forms) in embryos led to a complete loss of methylation at the majority of differentially methylated regions (DMRs) [25]. Thus, Dnmt1 alone is sufficient to maintain methylation imprints during cleavage. Considering the evidence available, we can speculate that maternal Dnmt1 maintains methylation imprinting at the one-cell stage and that zygotically expressed Dnmt1s maintains methylation imprints from the second cell cycle onward.

Although we do not know the mechanism by which Dnmt1 maintains methylation imprints against active and passive genome-wide demethylation in preimplantation embryos, we can confirm that Dnmt1 is present at very low levels in the nuclei of mouse MII oocytes and preimplantation embryos [23], [24], [25]. The first step of SCNT is enucleation, during this step, the nucleus and a small amount of the surrounding cytoplasm are removed from MII oocytes. Because maternal Dnmt1, the enzyme that maintains methylation imprints in MII oocytes, is localized in the nucleus, we hypothesized that enucleation result in loss of maternal nuclear Dnmt1, leading to subsequent failure to maintain methylation imprints in the cloned embryos derived from these oocytes.

Here, we investigate whether methylation imprints are maintained in SCNT-derived preimplantation embryos and how or to what extent maternal nuclear Dnmt1 contributes to the loss of methylation imprints in cloned embryos. We attempted to produce cloned embryos with nonenucleated oocytes to determine whether methylation imprints were rescued because such a result would provide clear evidence for the presence of maternal nuclear Dnmt1.

## Results

### Demethylation of imprinted genes in cloned preimplantation embryos

**Figure 1:**
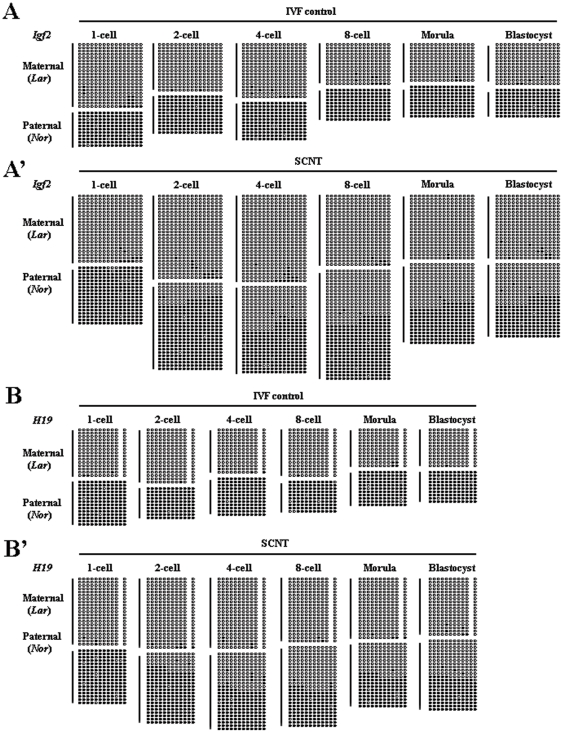
Methylation status of the DMRs at various stages of IVF and cloned preimplantation embryos. (A) Methylation status of the *Igf2* gene in IVF preimplantation embryos. All stages of preimplantation embryos exhibited hemimethylation at this locus, indicating the maintenance of methylation imprints. (A′) Methylation status of the *Igf2* gene in SCNT-derived preimplantation embryos. Demethylation occurred during the first two cell cycles, and in each cycle, approximately one-quarter of the normal methylation was lost. (B) Methylation status of the *H19* gene in IVF preimplantation embryos. Hemimethylation also occurred in all stages of preimplantation embryos. (B′) Methylation status of the *H19* gene in SCNT-derived preimplantation embryos. Demethylation also occurred in the first two cell cycles; in each cell cycle, approximately one-quarter of the normal methylation was lost. (Lar) Large White-derived allele; (Nor) Northeast Min-derived allele.

To determine whether the DNA methylation of imprinted genes was properly reprogrammed during SCNT, we examined the DNA methylation status of imprinted genes at various stages of cloned preimplantation embryo development using bisulfite sequencing. *In vitro* fertilized (IVF) embryos served as controls. Based on previous studies, we selected DMR2 of *Igf2* and ICR3 of *H19* for analysis [26], [27]. We confirmed the methylation patterns of both regions by analyzing DNA methylation status in sperm and oocytes. Both DMRs were differentially methylated between the paternal and maternal genomes (Fig. S1). To determine whether methylation imprints were maintained after fertilization, as seen in mice and humans, we first examined the methylation status of imprinted genes in IVF preimplantation embryos. To distinguish the paternal and maternal alleles, we introduced SNPs into the DMRs of IVF embryos by obtaining gametes from pigs of different genetic backgrounds (Fig. S2). Preimplantion IVF embryos exhibited hemimethylation at both loci during all stages of development examined (Fig. 1A, B), indicating the maintenance of methylation imprints at these loci. We also examined the methylation status of *in vivo* blastocysts, which also exhibited hemimethylation at both loci (Fig. S3). These results clearly excluded the possibility that imprinting disruption was caused by *in vitro* cultivation of the embryos. We then examined the methylation status of imprinted genes in cloned preimplantation embryos. In cloned preimplantation embryos, both genes (*Igf2* and *H19*) were demethylated (Fig. 1A′, B′). In particular, demethylation of both genes occurred at the one- and two-cell stages, after which methylation imprints were maintained until the blastocyst stage. Furthermore, at each stage of the first two-cell cycles, both DMRs lost approximately one-quarter of their normal methylation (Fig. 1A′, B′), suggesting that with each cell cycle the genes had undergone the same or nearly the same loss of DMR methylation.

**Figure 2:**
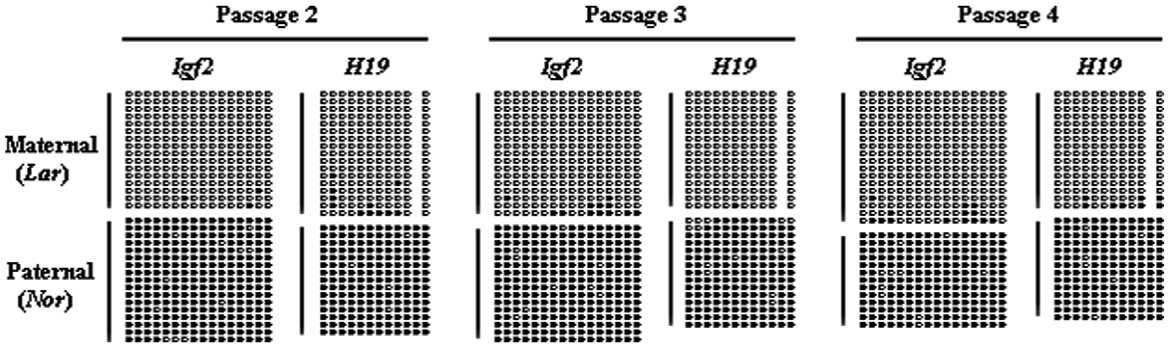
Methylation status of DMRs in different passages of donor fibroblasts. Hemimethylation occurred at both loci in passage 2 (left), passage 3 (middle) and passage 4 (right) donor fibroblasts, indicating the maintenance of methylation imprints during cell culture. (Lar) Large White-derived allele; (Nor) Northeast Min-derived allele.

Although we excluded that imprinting disruption could be caused by in vitro embryo cultivation, we wanted to determine whether it could be caused by donor cell cultivation. Donor cells used for SCNT were passaged *in vitro* (passages 2 through 4) to assess the possibility that the disruption of imprinting was brought about by donor cell cultivation. We then examined the methylation status of the imprinted genes at various passages. The methylation imprints of both *Igf2* and *H19* were unchanged in fibroblasts at passages 2 through 4 (Fig. 2), indicating that the disruption of methylation imprints was not due to donor cell cultivation.

### Demethylation is dependent on DNA replication

**Figure 3:**
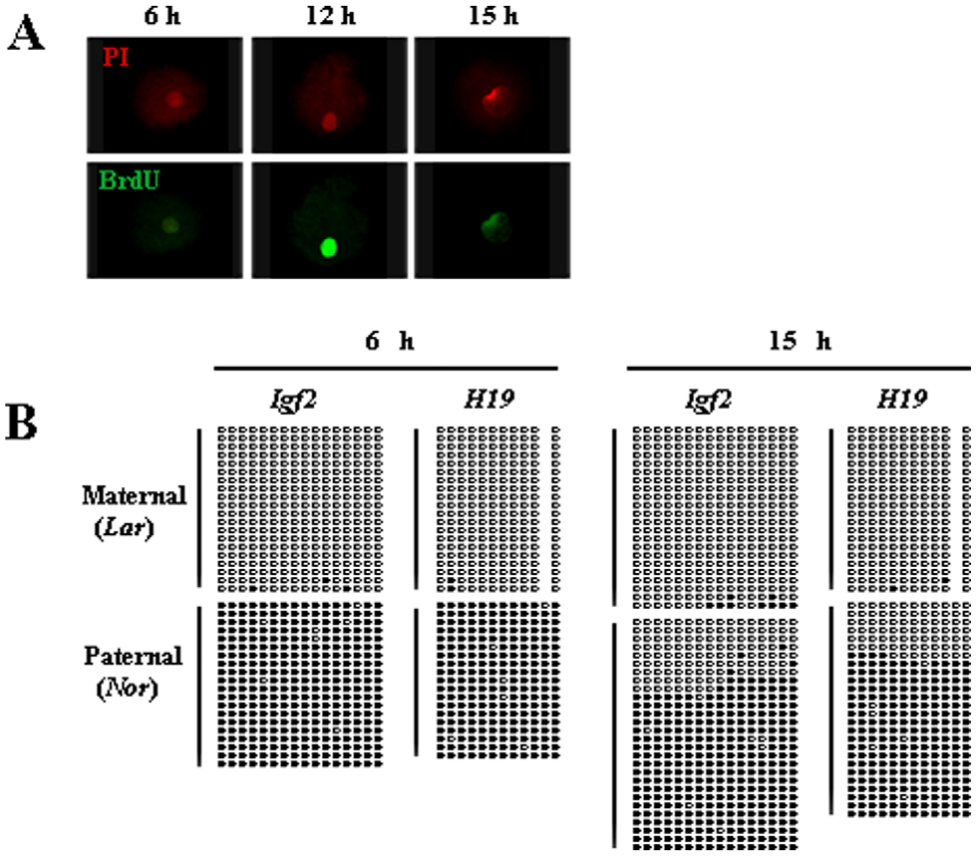
Demethylation is dependent on DNA replication. (A) Detection of DNA replication at various times in one-cell SCNT embryos by BrdU labeling. (Left) At 6 h post-activation, a few embryos were labeled by BrdU and were a dark color, indicating the beginning of DNA replication. (Middle) At 12 h post-activation, most embryos were labeled by BrdU and were a bright color, indicating the peak of DNA replication. (Right) At 15 h post-activation, few embryos were labeled by BrdU and were dark in color again, indicating near completion of DNA replication. (B) Methylation status of DMRs at 6 and 15 h in one-cell SCNT embryos. (Left) At 6 h post-activation, methylation was unchanged compared with donor cells. (Right) At 15 h post-activation, demethylation had occurred, and each DMR lost about one-quarter of its normal methylation. (Lar) Large White-derived allele; (Nor) Northeast Min-derived allele.

To determine whether demethylation was the result of failure to maintain methylation, we examined the period during which DNA replication and demethylation occur in one-cell-stage cloned embryos. We used BrdU labeling to analyze the behavior of DNA at 6, 9, 12, 15, and 18 h after embryo activation. Only a few embryos were labeled by BrdU at 6 h and these exhibited a dark color, indicating the initiation of DNA replication; most embryos were labeled at 12 h and exhibited a bright color, indicating the peak of DNA replication; few embryos were labeled at 15 h and these were also dark in color, indicating that DNA replication was close to completion; and no embryos were labeled at 18 h, indicating that DNA replication was complete (Fig. 3 A, Table 1). Together, these results suggested that DNA replication of one-cell cloned embryos occurred at approximately 6 to 15 h after embryo activation. We then analyzed the DNA methylation status of imprinted genes at 6 and 15 h after embryo activation. Methylation imprints were unchanged at 6 h, compared with donor cells (Fig. 3 B, Fig. 2). However, demethylation occurred from 6 to 15 h post-activation, which corresponded to the period of DNA replication (Fig. 3 B). Methylation imprints were unchanged again from 15 h to the beginning of the two-cell stage. (Fig. 3 B, Fig. 1 A′ and B′). These results indicated that demethylation was the result of failure to maintain methylation imprints, which was dependent on DNA replication.

**Table 1:** Examination of DNA replication in one-cell stage SCNT embryos by BrdU labeling.

Time after activation	6 hr	9 hr	12 hr	15 hr	18 hr
Total no. of embryos examined	36	71	62	68	33
Total no. (%) of embryos labeled	2 (6)	22 (31)	50 (81)	11 (16)	0 (0)

### Expression of Dnmt1 in oocytes and preimplantation embryos

**Figure 4:**
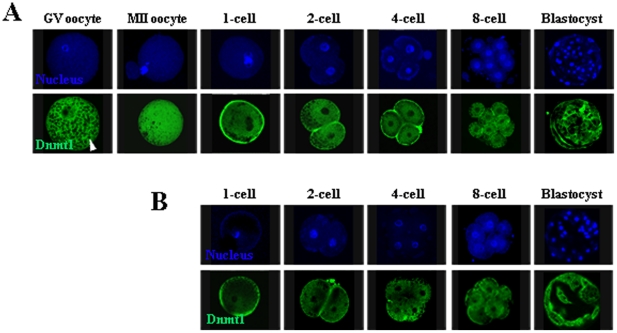
Expression and subcellular localization of Dnmt1 in oocytes and preimplantation embryos. (A) Immunostaining of GV oocytes, MII oocytes, and IVF preimplantation embryos with an anti-Dnmt1 antibody recognizing both maternal and zygotic Dnmt1. Dnmt1 signals (green) were mainly detected in the ooplasm and the cytoplasm of preimplantation embryos. (B) Dnmt1 also localized cytoplasmically in SCNT-derived preimplantation embryos. The cell nucleus was counterstained with Hoechst 33342 (blue). Arrowhead indicates lipid droplets.

The only known functional maintenance methyltransferase is Dnmt1. To investigate the role of Dnmt1 in the maintenance of methylation imprints in preimplantation embryos, we first examined its expression and subcellular localization in oocytes and preimplantation embryos. We observed strong Dnmt1 signals in the cytoplasm of oocytes and IVF preimplantation embryos at all stages of development (Fig. 4 A), consistent with reports in mice [21], [22]. We then examined the status of Dnmt1 in preimplantation embryos obtained from SCNT, and the enzyme was also localized cytoplasmically (Fig. 4 B). Thus, the vast majority of the Dnmt1 proteins were localized to the cytoplasm of both IVF- and SCNT-derived cloned preimplantation embryos. However, we do not exclude the possibility that very low levels of Dnmt1 were present in the nuclei of oocytes and preimplantation embryos.

### Dnmt1 is required for the maintenance of methylation imprints in preimplantation embryos

**Figure 5:**
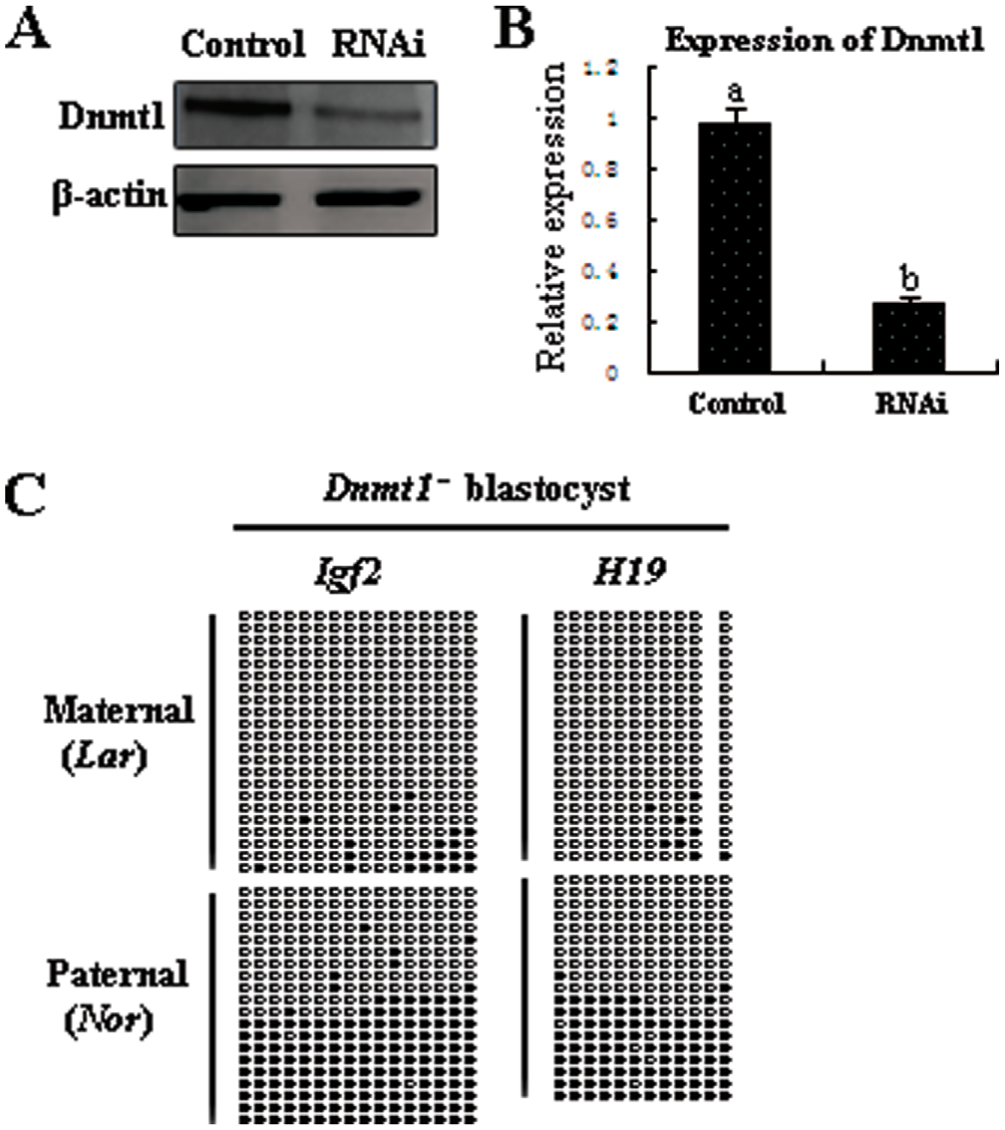
Loss of methylation at DMRs in Dnmt1-deficient blastocysts. RNAi efficiency was confirmed by Western blot (A) and quantitative real-time PCR (B). (C) A partial reduction of methylation at DMRs was found in day 7 Dnmt1-deficient blastocysts, indicating that Dnmt1 is required for the maintenance of methylation imprints. (Lar) Large White-derived allele; (Nor) Northeast Min-derived allele.

To directly investigate whether Dnmt1 is involved in the maintenance of methylation imprints in preimplantation embryos, we studied the methylation status of imprinted genes in RNAi-mediated *Dnmt1* knockdown blastocysts. We injected *Dnmt1* siRNA or negative control siRNA into GV stage oocytes. After culturing for 42 h, we collected the MII stage oocytes and examined the RNAi efficiency by quantitative real-time PCR and Western blotting. Expression of Dnmt1 transcripts and protein in siRNA-injected MII oocytes were significantly reduced, compared with controls (control siRNA injection), indicating successful *Dnmt1* knockdown by RNAi (Fig. 5 A, B). We then carried out IVF with the *Dnmt1* knockdown MII oocytes. After culturing for 7 d, we collected the blastocysts and examined the methylation status of the imprinted genes. The blastocysts exhibited a partial reduction of methylation at both DMRs (Fig. 5 C). The observed methylation defects were similar to those produced by SCNT (Fig. 1 A′, B′). These results suggested that Dnmt1 is required for the maintenance of methylation imprints in porcine preimplantation embryos.

### Imprinting rescued by SCNT with nonenucleated MII oocytes

**Figure 6:**
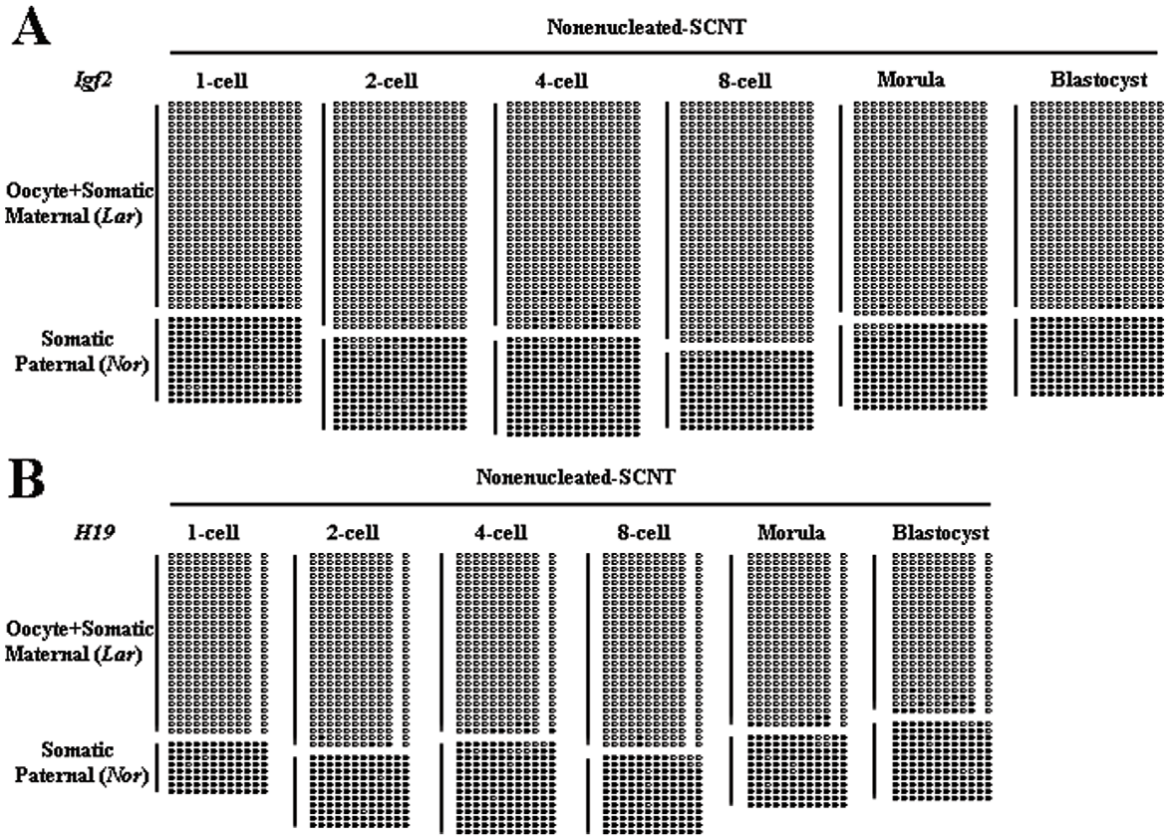
Rescue of methylation imprints by SCNT with nonenucleated MII oocytes. Almost all of the somatic paternal original alleles were fully methylated at both the *Igf2* (A) and *H19* (B) DMRs, indicating that imprinting was rescued by the nuclei of MII oocytes. Oocyte+Somatic Maternal (Lar) indicates oocyte and somatic maternal original alleles, both of which are Large White-derived alleles. Somatic Paternal (Nor) indicates somatic paternal alleles, which are Northeast Min-derived alleles.

The above results suggested that, although we did not observe Dnmt1 signals in the nuclei of oocytes and preimplantation embryos (Fig. 4), there must be a small amount of nuclearly localized Dnmt1 protein that maintains methylation imprints. Because enucleation is an essential step of SCNT, we suspected that this low level of protein is lost during this step. To assess this possibility, we constructed embryos with somatic cells and nonenucleated MII oocytes and studied the methylation status of imprinted genes in preimplantation embryos. To exclude the effects of micromanipulation, we also aspirated a small amount of cytoplasm in the nonenucleated group. The embryos constructed with nonenucleated oocytes were developmentally normal ability compared with the enucleated group (Table 2). Bisulfite sequencing analysis showed that almost all of the somatic paternal original alleles were fully methylated at both the *Igf2* (Fig. 6 A) and *H19* (Fig. 6 B) DMRs, indicating that imprinting was rescued by the nuclei of the MII oocytes. These results suggested that the small amount of Dnmt1 that maintained methylation imprints was localized to the nuclei of the MII oocytes.

**Table 2:** *In vitro* development of pig zygotes after SCNT with nonenucleated MII oocytes.

Type of pig oocytes	Enucleated	Nonenucleated
Total no. of oocytes examined	243	285
Total no. (%) of oocytes cleaved	171 (70)	189 (66)
Total no. (%) of oocytes forming blastocysts	62 (26)	68 (24)

The values in parentheses represent the percentages of the embryos cleaved at 48 h and forming blastocysts at 7 days after SCNT.

### Detection of zygotic Dnmt1 in preimplantation embryos

**Figure 7:**
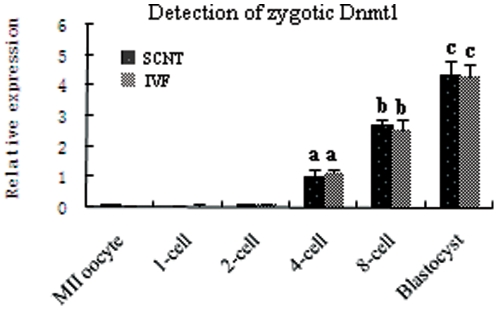
Detection of zygotic Dnmt1 in oocytes and preimplantation embryos by quantitative real-time PCR. Zygotic Dnmt1 transcripts were almost undetectable in oocytes and one- and two-cell stage embryos. However, the expression of zygotic Dnmt1 was significantly increased from the four-cell stage of both IVF and cloned preimplantation embryos. Thus, zygotic Dnmt1 was synthesized from the third cell cycle.

Demethylation of imprinted genes occurred mainly in the first two cell cycles of cloned preimplantation embryos, after which methylation imprints were maintained until the blastocyst stage. Thus, we suspected that zygotic Dnmt1 may be synthesized from the third cell cycle, which subsequently maintains the methylation imprints. To determine whether zygotic Dnmt1 was synthesized from the four-cell stage, we examined the expression levels of Dnmt1s in preimplantation embryos. The major form of zygotic Dnmt1 is Dnmt1s, which is localized in the nucleus of preimplantation embryos. Dnmt1s is different from Dnmt1o (the major form of maternal Dnmt1), which is localized in the cytoplasm of preimplantation embryos [21], [22], [28]. Strong Dnmt1 signals were only observed in the cytoplasm of preimplantation embryos (Fig. 4), indicating that the major form of Dnmt1 expressed in preimplantation embryos was Dnmt1o. We did not detect Dnmt1s by immunoblotting cell lysates from preimplantation embryos (data not shown); we inferred that the presence of the abundant Dnmt1o would interfere with the detection of Dnmt1s. Thus, we attempted to detect Dnmt1s in preimplantation embryos by quantitative real-time PCR. Because *Dnmt1o* and *Dnmt1s* transcripts use the same exons except for exon-1 (Fig. S4), we can distinguish between them using exon-1-specific primers [28]. Quantitative real-time PCR analysis showed that Dnmt1s expression was significantly increased beginning at the four-cell stage of both IVF and cloned preimplantation embryos (P<0.05). However, Dnmt1s transcripts were almost undetectable in oocytes and in one- and two-cell stage embryos (Fig. 7). These results indicated that zygotic Dnmt1 was synthesized beginning in the third cell cycle and might maintain methylation imprints in cloned preimplantation thereafter.

## Discussion

The major conclusion of this study is that the unfaithful maintenance of methylation imprints in cloned preimplantation embryos is a result of the loss of maternal nuclear Dnmt1 during SCNT. Our data also indicate that zygotic Dnmt1 is expressed beginning at the four-cell stage and may maintain the methylation imprints in SCNT embryos thereafter. It is noteworthy that methylation imprints were also disrupted in nuclear transfer-derived embryonic stem cells (ntESCs), which are derived from the inner cell mass (ICM) of cloned blastocysts [29], [30]. In fact, imprinting problems also occur in cloned mice and bovine embryos [15], [16], [31], [32], indicating that this is a general, rather than individual, characteristic of SCNT. Although Inoue et al. (2002) found normal allele-specific expression of seven imprinted genes in cloned mouse embryos, their expression in placenta was significantly reduced [15]. We suggest the following as the simplest explanation: cells with imprinting errors may be incorporated into the trophectoderm, while cells without or almost without imprinting errors are incorporated into the ICM. This idea can also explain why a small proportion of cloned embryos successfully develop to term without imprinting problems.

We further showed, by BrdU labeling, that demethylation is dependent on DNA replication, indicating that demethylation is the result of unfaithful maintenance of methylation. The only known functional maintenance methyltransferase is Dnmt1, which exhibits a high affinity for hemimethylated DNA [33], [34]. We further showed that inactivation of *Dnmt1* in preimplantation embryos by RNAi-mediated knockdown caused a partial reduction of methylation at both the *Igf2* and *H19* DMRs. However, no clear Dnmt1 signal was detected in the nuclei of oocytes or preimplantation embryos. Thus, we can infer that Dnmt1 is present at very low levels in the nuclei of oocytes and preimplantation embryos. This result is consistent with mouse studies showing that Dnmt1 is detectable by specific antibodies in the nuclei of oocytes and preimplantation embryos [23], [24].

Methylation imprints were rescued by SCNT with nonenucleated MII oocytes, indicating that Dnmt1 present in the nuclei of MII oocytes maintains the methylation imprints of donor cells. Furthermore, we also performed a sham operation by aspirating a small amount of cytoplasm in the nonenucleation group, which showed that the demethylation is neither caused by micromanipulation nor by a reduction of cytoplasm, but rather, is a direct consequence of the absence of maternal nuclear Dnmt1. Zygotic Dnmt1 transcripts were almost undetectable at the one- and two-cell stages but became detectable starting at the four-cell stage, so the transcripts are considered of zygotic origin. Furthermore, because zygotic Dnmt1 (Dnmt1s) is present in the nucleus, we infer that it maintains methylation imprints beginning in the four-cell stage of cloned embryos. Ratnam et al. (2002) also found increased zygotic Dnmt1 transcripts from the two-cell stage in mouse embryos [22]. Considering the available evidence, a likely scenario is that maternal nuclear Dnmt1 maintains the methylation imprints during the first two cell cycles and that zygotic Dnmt1 is expressed from the four-cell stage and maintains the methylation imprints thereafter. During SCNT, maternal nuclear Dnmt1 is lost by enucleation, leading to failure to maintain methylation imprints of one- and two-cell stage cloned embryos. Zygotic Dnmt1 is synthesized from the four-cell stage and maintains the methylation imprints of cloned embryos thereafter.

Importantly, we did not observe the previously reported reestablishment of methylation imprints in porcine preimplantation embryos [35]. This difference may be a result of the different methods used in the previous study (Park et al. did not distinguish each of the parental alleles by allele-specific polymorphisms). Thus, the porcine methylation imprints observed are in accord with those in mice and humans, which are maintained after fertilization and in preimplantation embryos of all stages [8], [36]. For the successful development of a cloned embryo, proper genomic imprinting of the donor nucleus is clearly required [11], [37]. The methylation marks of some imprinted genes can be affected by culture conditions [38], [39]. However, we did not observe methylation imprint changes during cell or embryo culture. Thus, the possibility that the observed alterations of methylation imprints are triggered by culture conditions can be excluded.

In conclusion, our results indicate that the loss of maternal nuclear Dnmt1 during SCNT significantly contributes to unfaithful maintenance of methylation imprints in cloned preimplantation embryos. At present, we do not know how Dnmt1 enters the nucleus and maintains methylation imprints against the active and passive genome-wide demethylation that occurs in preimplantation embryos. We speculate that some unknown molecular mechanism may recruit Dnmt1 specifically to the DMRs. Understanding the mechanism underlying the unfaithful maintenance of methylation imprints in cloned preimplantation embryos would provide a basis for the improvement of reproductive cloning and the generation of ntESCs.

## Materials and Methods

### Ethics statement

Porcine handling was conducted in accordance with policies promulgated by the Ethics Committee of the Northeast Agriculture University. The institute does not issue a number to any animal study, but there is an ethical committee to guide animal use. Each study requires the permit to use animals from the committee. The only used materials derived from animals were porcine ovaries and sperm, which were obtained from DaZhongRouLian slaughterhouse, a local slaughterhouse in Harbin, P.R. China.

### Porcine

To introduce single nucleotide polymorphisms (SNPs) into the DMRs, porcine gametes and cells with different genetic backgrounds were used. Spermatozoa to be used for *in vitro* fertilization (IVF) were obtained from Northeast Min Pigs (a local breed), while oocytes to be used for IVF or SCNT were obtained from Large White Pigs. The fibroblasts used for donor cells were obtained from crosses between Northeast Min males and Large White females.

### Somatic cell nuclear transfer

The procedure for porcine SCNT has been described previously [26]. Briefly, micromanipulations were performed in manipulation medium containing 7.5 µg/ml cytochalasin B. After removal of the MII plate, a fibroblast was introduced into the perivitelline space of an enucleated oocyte. The oocytes were activated by two direct pulses of 120 V/mm for 30 µsec in fusion medium. Fused eggs were then cultured in Porcine Zygote Medium-3 (PZM-3).

### *In vitro* fertilization

Freshly ejaculated sperm-rich fractions were collected from fertile boars, and following a short incubation at 39°C, the semen was resuspended and washed three times in Dulbecco's Phosphate Buffered Saline (DPBS) supplemented with 0.1% (w/v) bovine serum albumin (BSA) by centrifugation at 1500×g for 4 min. The spermatozoa concentration was measured using a hemocytometer, and the proportion of motile sperm was determined. The spermatozoa were diluted with modified Tris-buffered medium (mTBM) to an optimal concentration. Cumulus-free matured oocytes were washed three times in mTBM. Approximately 30 oocytes were inseminated in 50-µl drops of mTBM at a final sperm concentration of 3×10^5^ cells/ml for 5 h.

### Oocyte and embryo collection

*In vitro* maturation (IVM) was performed according to established methods [26]. Briefly, porcine ovaries were collected from a local slaughterhouse and kept in saline at 32–37°C. Antral follicles (3–5 mm in diameter) were aspirated with an 18-gauge needle. Aspirated oocytes with an evenly granulated cytoplasm and three uniform layers of compact cumulus cells were selected and cultured in four-well plates containing 500 µl of TCM199 (Gibco) based medium for 42 h. At the end of IVM, the cumulus cells of cumulus oocyte complexes (COCs) were removed by vortexing for 2 m in TCM199 based medium supplemented with 0.5% hyaluronidase (Sigma).

Oocytes at the germinal vesicle (GV) and MII stages and preimplantation embryos were collected. For preimplantation embryos, one-cell, two-cell, four-cell, 8-cell, morula and blastocyst stage embryos were collected at 6 h, 24 h, 48 h, 72 h, 120 h and 168 h post-activation, respectively. To exclude the possible contamination of remaining cumulus cells or sperm, the zona pellucida was removed by treatment with warm Tyrode's acidic solution for 20 sec.

### Bisulfite sequencing

Bisulfite treatment of DNA was performed with an EZ DNA Methylation Kit (Zymo Research). Briefly, genomic DNA was isolated from 1×10^3^ sperm cells, 100 MII oocytes, 50 one- to four-cell stage embryos, 10 eight-cell stage embryos, and five embryos at later stages. Genomic DNA was then denatured in 0.3 M NaOH for 10 min at 37°C, treated with 9 M sodium bisulfite for 1 h at 70°C, collected using a microcolumn and desulphonated with 0.3 M NaOH. After the desulphonation, DNA was eluted with 10 to 20 µl of elution buffer. The DMRs of interest were amplified by PCR and subjected to sequence analysis. The primer sequences used were described previously [26], except for those of the *Igf2* DMR (GenBank accession no. AY044828.1, forward, 5′ -GTTAGGGGGGGGTTTGGTTTTTTAG- 3′; reverse, 5′ - CTCCCCTTAATCCTATAAAACTTCC- 3′; forward nested, 5′ -GGTTTTTTGGTTTAGAGGAGAT- 3′; reverse nested, 5′ -CTATAAAACTTCCAAACAAACC- 3′).

### BrdU labeling

DNA replication in one-cell stage embryos derived from SCNT was evaluated after incubating the embryos with 5-bromo-2′-deoxyuridine (BrdU). The embryos were labeled with 100 µM BrdU for 1 h beginning at 6, 9, 12, 15 and 18 h post-activation. Embryos were then washed in phosphate-buffered saline-polyvinyl alcohol (PBS-PVA) and fixed for 40 min in 4% paraformaldehyde containing 1% TritonX-100. After washing three times with BSA-PBS, DNA was denatured by incubating the embryos in 4 M HCl for 1 h. The acid was neutralized in 0.1 M Tris-HCl buffer (pH 8.5) for 15 min. After washing three times with BSA-PBS, the embryos were incubated with an anti-BrdU monoclonal antibody conjugated with Alexa Fluor 488 (1∶100, Molecular Probes) for 1 h at room temperature. Finally, after washing three times, the embryos were observed by fluorescence microscopy.

### RNA interference

Chemically synthesized 21-nt siRNA duplexes were commercially obtained (China GenePharma). *Dnmt1* siRNAs were microinjected into the cytoplasm of GV oocytes to deplete Dnmt1 (GenBank accession number: NM_001032355). Approximately 10 pl of 20 µM siRNA was used per oocyte with the following sequence: 5′-GGAAGAAGAUGAUAAAGAATT-3′. The same amount of negative control siRNA was injected as a control. After microinjection, the GV oocytes were cultured for 42 h until maturation and were subsequently subjected to IVF.

### Immunofluorescence and confocal microscopy

Oocytes or embryos were fixed with 4% paraformaldehyde/PBS (pH 7.4) for at least 40 min followed by permeabilization with 1% Triton X-100 at room temperature for 30 min, blocking in 1% BSA-supplemented PBS for 1 h and then incubating with a rabbit anti-Dnmt1 antibody (Santa Cruz; 1∶200) overnight at 4°C. After three washes with PBS containing 0.1% Tween 20 and 0.01% Triton X-100 for 5 min each, oocytes or embryos were labeled with 1∶200 FITC-conjugated IgG for 1 h at room temperature. After washing in PBS containing 0.1% Tween 20 and 0.01% Triton X-100, the oocytes or embryos were co-stained with Hoechst33342 (10 µg/ml in PBS). Finally, the oocytes or embryos were mounted on glass slides and examined with a confocal laser scanning microscope (Zeiss LSM 510 META, Germany).

### Immunoblotting analysis

Porcine oocytes at the GV or MII stage injected with or without *Dnmt1* siRNA were collected in sodium dodecyl sulfate (SDS) sample buffer and boiled for 5 min. Immunoblotting was performed as described previously [40]. Briefly, the proteins were separated by sodium dodecyl sulfate polyacrylamide gel electrophoresis (SDS-PAGE) and then electrophoretically transferred to polyvinylidene fluoride membranes. Following transfer, the membranes were blocked in Tris-buffered saline Tween-20 (TBST) containing 5% non-fat milk for 2 h followed by incubation overnight at 4°C with an anti-Dnmt1 antibody (Santa Cruz) at dilutions of 1∶500. After washing in TBST, the membranes were incubated for 1 h at 37°C with 1∶1000 horseradish peroxidase (HRP)-conjugated IgG. To detect β-actin, the membranes were washed in washing buffer (100 mM β-mercaptoethanol, 20% SDS, and 62.5 mM Tris, pH 6.7) for 30 min at 55°C. Then β-actin was assayed on the same membrane using an anti-β-actin antibody (1∶1000) and HRP-conjugated IgG. Finally, the membranes were detected using an enhanced chemiluminescence detection system (Amersham, Piscataway, NJ).

### Nuclear transfer with nonenucleated oocytes

All of the procedures were performed as for enucleated oocytes, except for the enucleation step. Oocytes were held with the first polar body (PB1) at the 3 o'clock position, and then PB1 was removed. To exclude the effects of micromanipulation, a small amount of cytoplasm was aspirated from the MII oocytes. Before aspirating, the oocyte was rotated (approximately 90°) to avoid damaging the nucleus. A donor cell was then transferred to the perivitelline space at approximately the 3 o'clock position.

### Quantitative real-time PCR

Total RNAs were isolated from the oocytes and embryos using an RNeasy mini kit (Qiagen). First-strand cDNAs were synthesized with a SuperScript™ III first-strand synthesis system (Invitrogen) according to the manufacturer's instructions. For quantitative real-time PCR, reactions were performed using SYBR Premix Ex Taq™ (TaKaRa) and a 7300 Real-Time PCR System (Applied Biosystems). The data were obtained from three individual trials for each sample. The primers used for real-time PCR were: *Dnmt1*; 5′-CTGTGCTGGGATAGATA-3′ and 5′-AGATGACCTTCACTTTGCT-3′; *β-actin*; 5′-CCTGTACGCCTCTGGCCGCA-3′ and 5′-GGACTTCGAGCAGGAGATGG-3′. The data were analyzed using the 2^−ddCt^ method with β-actin as an internal control.

### Statistical analysis

Data (mean ± SE) were collected from at least three replicates per experiment and analyzed by ANOVA using SPSS software (SPSS Inc, Chicago, IL) followed by a student-Newman-Keuls test. P<0.05 was considered statistically significant.

## Supporting Information

Figure S1 **Methylation status of DMR2 of** ***Igf2*** **and ICR3 of** ***H19*** **in porcine gametes.** Lollipops represent all examined CG dinucleotides. Black and white circles represent methylated and unmethylated CpGs, respectively. Each line represents a separate clone.
(TIF)

Figure S2 **Sequence chromatograms of DMRs amplified from Large White and Northeast Min pigs.** The DMR2 of *Igf2* amplified from oocytes of Large White (A) and sperm of Northeast Min (A′), indicating the position of a C/G single nucleotide polymorphism; ICR3 of *H19* amplified from oocytes of Large White (B′) and sperm of Northeast Min (B′), indicating the position of an A/G single nucleotide polymorphism.
(TIF)

Figure S3 **Methylation status of DMRs in** ***in vivo*** **blastocysts.** Hemimethylation also occurred at both loci, as seen in that of IVF blastocysts.
(TIF)

Figure S4 **Distinguishing** ***Dnmt1o*** **and** ***Dnmt1s*** **transcripts based on exon 1.** *Dnmt1o* and *Dnmt1s* utilize the same exons, except for exon 1, resulting in unique sequences at their 5′ ends. Thus, we can distinguish them by exon-1-specific primers.
(TIF)
