# Supplementary figures and images for: Unfaithful Maintenance of Methylation Imprints Due to Loss of Maternal Nuclear Dnmt1 during Somatic Cell Nuclear Transfer
Source: PLoS One. 2011 May 20;6(5):e20154. doi: 10.1371/journal.pone.0020154 (PMC3098883; doi:10.1371/journal.pone.0020154)

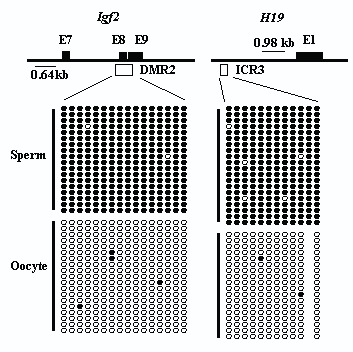

Supplement: Figure S1 — Methylation status of DMR2 of Igf2 and ICR3 of H19 in porcine gametes. Lollipops represent all examined CG dinucleotides. Black and white circles represent methylated and unmethylated CpGs, respectively. Each line represents a separate clone. (TIF) [file pone.0020154.s001.tif]

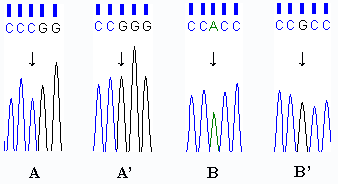

Supplement: Figure S2 — Sequence chromatograms of DMRs amplified from Large White and Northeast Min pigs. The DMR2 of Igf2 amplified from oocytes of Large White (A) and sperm of Northeast Min (A′), indicating the position of a C/G single nucleotide polymorphism; ICR3 of H19 amplified from oocytes of Large White (B′) and sperm of Northeast Min (B′), indicating the position of an A/G single nucleotide polymorphism. (TIF) [file pone.0020154.s002.tif]

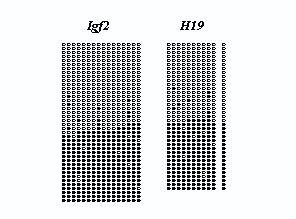

Supplement: Figure S3 — Methylation status of DMRs in in vivo blastocysts. Hemimethylation also occurred at both loci, as seen in that of IVF blastocysts. (TIF) [file pone.0020154.s003.tif]

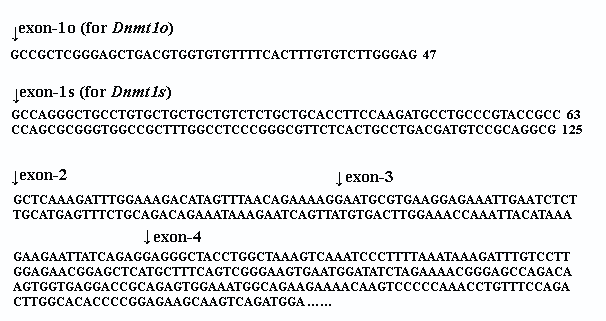

Supplement: Figure S4 — Distinguishing Dnmt1o and Dnmt1s transcripts based on exon 1. Dnmt1o and Dnmt1s utilize the same exons, except for exon 1, resulting in unique sequences at their 5′ ends. Thus, we can distinguish them by exon-1-specific primers. (TIF) [file pone.0020154.s004.tif]
